# Simultaneous EEG-fMRI reveals attention-dependent coupling of early face processing with a distributed cortical network

**DOI:** 10.1016/j.biopsycho.2017.12.002

**Published:** 2018-02

**Authors:** Mareike Bayer, Michael T. Rubens, Tom Johnstone

**Affiliations:** a Berlin School of Mind and Brain, Humboldt-Universität zu Berlin, Berlin, Germany; b Centre for Integrative Neuroscience and Neurodynamics, School of Psychology and Clinical Language Sciences, The University of Reading, Reading, UK

**Keywords:** EEG, fMRI, Face processing, Attention, Stroop

## Abstract

The speed of visual processing is central to our understanding of face perception. Yet the extent to which early visual processing influences later processing in distributed face processing networks, and the top-down modulation of such bottom-up effects, remains unclear. We used simultaneous EEG-fMRI to investigate cortical activity that showed unique covariation with ERP components of face processing (C1, P1, N170, P3), while manipulating sustained attention and transient cognitive conflict employing an emotional face-word Stroop task.

ERP markers of visual processing within 100 ms after stimulus onset showed covariation with brain activation in precuneous, posterior cingulate gyrus, left middle temporal gyrus, left inferior frontal gyrus and frontal operculum, and a left lateral parietal-occipital cluster, illustrating the impact of early stage processing on higher-order mechanisms. Crucially, this covariation depended on sustained attentional focus and was absent for incongruent trials, suggesting flexible top-down gating of bottom-up processing.

## Highlights

• Distributed cortical activation to faces covaries with ERPs as early as 100 ms.
• Covariations depend on both sustained attention and trial-by-trial cognitive conflict.
• Top-down gating might apply to earlier visual processing stages than previously known.

## 1 Introduction

The speed of visual processing plays an important role in attempts to understand human visual perception in general, and face perception more specifically. Processing speed can provide evidence about the number of computational steps that are proposed by theories of visual perception (Thorpe, 2009). Investigating the time course of visual processing also allows for conclusions about the relative contributions of bottom-up and top-down influences on different stages of visual perception. Recent evidence suggests that categorical information can be extracted from visual input at very short latencies; reliable differences between faces and noise textures in single-trial ERP data were shown to occur as early as 90 ms after stimulus onset (Bieniek, Bennett, Sekuler, Rousselet, & Foxe, 2016). In a saccadic choice task (Crouzet, Kirchner, & Thorpe, 2010), reliable saccades toward faces (versus other objects) occurred from 100 ms after stimulus presentation, suggesting very fast extraction and utilization of categorical information. Recording intracranial field potentials, Liu, Agam, Madsen, & Kreiman (2009) demonstrated that the object category of visual stimuli can be decoded from single-trial data approximately 100 ms post stimulus-onset. The latter findings were interpreted as lending support to theories that assume a strong role of bottom-up and feed-forward processing in visual recognition (Liu et al., 2009). Even though there is little doubt that both bottom-up and top-down processes ultimately contribute to visual recognition, there is some controversy about their relative contributions, and particularly about the time course of bottom-up and top-down influences on perception (see Rousselet, Gaspar, Wieczorek, & Pernet, 2011).

A common means of investigating top-down modulation is the examination of task effects on visual perception. ERP research has shown that several top-down factors can influence activity in the visual cortex: Effects of spatial attention are reliably observed within 100 ms post stimulus-onset in the form of increased P1 amplitudes for attended versus unattended locations (Luck, Woodman, & Vogel, 2000), indicating increased sensory processing in the extrastriate visual cortex (Di Russo, Martínez, Sereno, Pitzalis, & Hillyard, 2002). Notably, recent research has demonstrated that even the primary visual cortex is susceptible to top-down modulation, including influences of attention, reward, and mood (Bayer et al., 2016; Proverbio, Del Zotto, & Zani, 2010; Rossi and Pourtois, 2012, Vanlessen et al., 2013). In contrast, the time course of task-related effects on object recognition remains a matter of debate, especially in the case of face perception (for discussion, see Rousselet et al., 2011).

A related question is the impact that early stage visual cortex processing has on subsequent processing in more anterior regions of the brain, including temporal, parietal and frontal cortical regions, and how such influences are affected by top-down processes. Behavioural and EEG evidence from tasks such as the attentional blink indicates that processing between 100 ms and 500 ms post-stimulus is particularly sensitive to attentional resource constraints and top-down effects (Folk, Leber, & Egeth, 2008; Shapiro, Raymond, & Arnell, 1997). In fMRI studies, activation in ventral visual object processing regions such as the fusiform face area (FFA) and parahippocampal place area (PPA) correlates with the magnitude of such behavioural effects (Lim, Padmala, & Pessoa, 2009). Highlighting the balance between bottom-up and top-down effects, the attentional blink is reduced when the visual stimulus is emotionally significant (De Martino, Kalisch, Rees, & Dolan, 2009), possibly through the influence of subcortical circuitry such as the amygdala and pulvinar (Vuilleumier & Driver, 2007).

Taken together, previous research provides evidence for rapid timing of both bottom-up visual processing and top-down modulation, both being evident within 100 ms after stimulus onset and involving a distributed network of cortical and subcortical brain regions. In the present study we investigated the influence of early information processing in the visual cortex on higher-order processing stages, and its top-down modulation by task-related attention, focusing specifically on the processing of faces. Using simultaneous EEG-fMRI, trial-by-trial ERP amplitudes were used to identify brain regions that showed significant and unique covariation with different visual processing stages, from very early perceptual processing in the striate and extrastriate visual cortex (as indicated by C1 and P1 components; Di Russo et al., 2002, Foxe and Simpson, 2002) to subsequent stages related to structural face encoding and stimulus evaluation (N170 and P3;, e.g. Eimer, 2000, Polich, 2007). Furthermore, the influence of sustained attention and trial-by-trial cognitive conflict on such covariation was examined.

Participants performed a modified Stroop task employing emotional face-word stimuli (Beall & Herbert, 2008; Etkin, Egner, Peraza, Kandel, & Hirsch, 2006). Happy, neutral and fearful faces were superimposed with the respective emotion words (congruent or incongruent); in separate blocks, participants were instructed to categorise the emotion of either face or word (Attend Face/Attend Word condition). The emotional face-word Stroop task is well-suited for our research question: First, since face and word are presented simultaneously and in a superimposed manner, it allows for the investigation of sustained top-down attentional focus on one of the two object categories during *identical* perceptual input. Second, contrasting congruent and incongruent face-word pairs provides insight into conflict processing, a mechanism at the intersection of bottom-up and top-down processing, which includes trial-by-trial conflict monitoring and resolution. Finally, although the processing of emotional stimuli *per se* was not the focus of this study (and the design would not have the power to examine emotion-specific effects), emotional faces were used as target stimuli to increase the significance of visual stimuli (Pourtois, Schettino, & Vuilleumier, 2013; Vuilleumier, 2015) and hence maximise the power of detecting early stage visual processing effects with trial-by-trial ERP-fMRI covariation analysis (e.g., Bieniek et al., 2016, Crouzet et al., 2010, Liu et al., 2009, Rousselet et al., 2011).

We predicted that i) early stage face processing as indexed by amplitudes in the P1 and possibly C1 time range would show trial-by-trial covariation with fMRI BOLD activation during the Attend Face condition throughout brain regions involved in face processing (Haxby, Hoffman, & Gobbini, 2000) and ii) these covariations would be modulated by task demands and would be reduced or absent in the Attend Word condition. For the congruent versus incongruent contrast, we predicted changes to ERP-BOLD covariation, though made no specific directional prediction.

## 2 Methods

The study was reviewed and approved by the University of Reading research ethics committee. Participants provided informed consent prior to taking part in the study.

### 2.1 Participants

18 participants took part in the study; data from 3 participants had to be excluded due to poor EEG data quality. The remaining sample of 15 participants (7 female participants) had a mean age of 23.4 years (SD = 3.8 years). All participants were right handed, had normal or corrected-to-normal vision and did not report any history of neurological or psychiatric disorders. Participants were recruited through the Undergraduate Research Panel and mailing lists; they received £20 or course credit for participation.

### 2.2 Stimuli and task

Stimuli consisted of 216 portraits of unfamiliar females and males with superimposed affective words. The portraits displayed three different facial expressions (fearful, happy, neutral; 71 stimuli per category). The affective words corresponding to these emotion categories were ‘afraid’, ‘happy’, and ‘calm’. All of the pictures were adopted from the Karolinska Directed Emotional Faces set (KDEF; Lundqvist, Flykt & Öhman, 1998). The superimposed words were positioned horizontally in the middle of the face, and presented in white colour. Faces and words were combined in two ways: In congruent trials, the word matched the facial expression (e.g., the word ‘happy’ superimposed on a happy facial expression). In incongruent trials, the word did not match the facial expression. The ratio of congruent and incongruent trials was 50%. Participants were instructed to perform an affective categorisation task on the stimuli by pressing the corresponding button on the button box. We manipulated attentional focus by presenting stimuli in two types of blocks: In half of the blocks, the affective categorisation was performed on the face while ignoring the word (Attend Face); in the other half of blocks, categorisation was performed on the words (Attend Word). Stimuli were presented in six blocks, each consisting of 36 trials and lasting approximately 5 min. The order of stimuli in each block was randomized; the order of Attention conditions (Attend Face/Attend Word) and the assignment of affective categories to response buttons were counterbalanced.

Two comparisons were of particular interest. For the effect of top-down attention on face processing, we compared Attend Face vs. Attend Word in the congruent condition. For the effect of conflict processing on face processing, we compared congruent vs. incongruent trials in the Attend Face condition.

### 2.3 Procedure

The experiment took place in the MRI facility in the Centre for Integrative Neuroscience and Neurodynamics at the University of Reading. After providing informed consent, participants were given information concerning the task and the procedure. After EEG preparation, participants performed 10 practice trials in order to practice the button assignments to affective categories.

The experiment employed an event-related paradigm programmed in Matlab (MathWorks) utilizing the Psychophysics Toolbox extensions (Brainard, 1997, Pelli, 1997). Stimuli were displayed using a Nordic Neuro Labs MRI-compatible goggle visual display system at 60 Hz on an 800 × 600 pixel screen, with a field of view of 30 × 23°. At the start of each trial, a fixation cross was presented in the centre of the screen, followed by a target stimulus displayed for 700 ms. Optseq 2 (Dale, 1999) was used to schedule the presentation of events by jittering the inter-trial interval in order to maximise statistical efficiency (Post stimulus delay parameters: minimum = 4.2 s, maximum = 10.5 s, step = 700 ms; Liu, 2004). Participants were asked to limit eye blinks to the presentation of the fixation cross, and to avoid blinking during stimulus presentation.

### 2.4 Data acquisition and preprocessing

#### 2.4.1 fMRI

Data were collected on a 3T Siemens Trio MRI scanner using the standard 12-channel head matrix coil (Siemens, Malvern, PA). Functional images were acquired with a t2*-weighted gradient echo EPI sequence (30 interleaved axial slices, phase encoding P to A, 3 × 3 mm voxels, slice thickness = 4 mm; 128 × 128 matrix; 230 mm FOV; TR = 2100 ms, TE = 31 ms, Flip Angle: 78°; 145 whole-brain volumes). After functional scans, a high resolution whole-brain structural image was acquired (MPRAGE sequence, 1 mm isotropic voxels, FOV = 160 × 256 × 256 mm, Flip Angle: 9°).

Data were processed with FSL (FMRIB’s Software Library, www.fmrib.ox.ac.uk/fsl). Brain extraction was performed using the FSL Brain Extraction Tool (Smith, 2002). Data was high-pass filtered with a cutoff frequency of 100 Hz and spatially smoothed with a 5 mm FWHM Gaussian kernel filter.

#### 2.4.2 EEG

Simultaneous to fMRI data acquisition, continuous EEG data were collected from 31 Ag-AgCl electrodes attached to an MRI-compatible EEG cap (BrainCap MR, BrainProducts) using an MRI-compatible amplifier system (BrainAmp MR plus 32, BrainProducts). An additional electrode was placed on the back of the participant, just left of the spinal column, for recording the electrocardiogram (ECG). Data were referenced to electrode Cz; AFz was used as ground. Electrode impedances where kept below 20 kΩ; data were recorded with a sampling rate of 5000 Hz. Optimal synchronisation of EEG recording and MRI slice acquisition was ensured by using a sync box (BrainProducts) which synchronises the scanner and EEG amplifier clocks.

Offline, MR gradient artefacts were removed using a modified version of the template subtraction algorithm (Allen, Josephs, & Turner, 2000) as implemented in BrainVision Analyzer 2 (BrainProducts). Gradient artefacts were identified using synchronized markers from the scanner and removed from continuous, baseline corrected data (using the whole artefact for baseline correction). Ballistocardiographic artefacts were identified using the ECG channel with a semiautomatic template matching procedure; correction was performed using a template subtraction approach based on a sliding window of 21 pulse intervals. After correction, data were down-sampled to 250 Hz and filtered using an IIR filter with a bandwidth from 0.031 to 40 Hz. In order to identify additional artefacts caused by eye blinks, eye movements and residual ballistocardiographic artefacts, we performed a restricted Infomax ICA (Bell & Sejnowski, 1995) and removed artefact components based on their topography and time course. Data was re-referenced to average reference and segmented into epochs from −100 ms to 800 ms relative to stimulus onset, and baseline-corrected using a 100 ms pre-stimulus baseline. Trials with activity exceeding ± 100 μV or voltage steps larger than 100 μV were marked as artefact trials (2.1% of trials). Further pre-processing and data analyses of segmented single-trial ERPs was performed using Matlab.

### 2.5 Data analyses

#### 2.5.1 Behaviour

Reaction times (RTs) for correct trials and percentage of correct responses were calculated for each experimental condition and analysed with a mixed effects General Linear Model (GLM) with the fixed factors Attention (face, word) and Congruency (congruent, incongruent), and Subject as random factor.

Of particular interest were RT differences between the Attend Face and Attend Word condition for congruent (i.e., identical) stimuli, which would indicate the direct effect of top-down attentional focus. In addition, differences between congruent and incongruent trials would indicate effects of stimulus conflict.

#### 2.5.2 fMRI

A first level GLM was modelled separately for each run of the experiment with separate regressors for congruent and incongruent trials. Regressors were created by convolving the temporal profile of each experimental condition with the double gamma haemodynamic response function in FSL. The contrast of interest compared activation in congruent vs. incongruent trials. Single-level contrasts for each run were combined across all runs in a second level fixed effects analysis for each participant. In this step, Attention (Attend Face, Attend Word), which had been manipulated between runs, was included as an additional contrast.

Registration to standard space was performed using FLIRT (Jenkinson & Smith, 2001). In a first step, the mean functional volume for each run was registered to the participant’s high resolution structural image using a BBR cost function. In a second step, the participant’s high resolution structural image was normalised to the Montreal Neurological Institute (MNI) template (MNI152_T1_2mm_brain) using a 12 DOF affine registration. Finally, these two transformations were combined for subsequent registration of the participant’s contrast images to MNI space. For higher-level analyses across participants, contrasts of interest (congruent vs. incongruent trials for the two Attention conditions) were entered into a mixed-effects GLM. To guard against spurious activations due to multiple voxelwise comparisons, all our whole-brain analyses were corrected using a voxelwise p < 0.01 and cluster thresholding based on Random Field Theory to ensure a corrected Familywise Error (FWE) of p < 0.05 (Friston, Worsley, Frackowiak, Mazziotta, & Evans, 1994; Nichols et al., 2017).

#### 2.5.3 ERPs

Amplitudes of the ERP components (C1, P1, N170, P3) were quantified at specific time windows and regions of interest (ROIs) according to their respective temporal and topographic distribution (see Fig. 3): C1 amplitudes were quantified as mean amplitudes in the time window of 60–80 ms after stimulus onset at centro-occipital electrodes Pz, POz and Oz; P3 amplitudes were calculated at the same electrodes in the time window of 300–400 ms. P1 amplitudes were calculated at occipital electrodes (O1, Oz, O2), and the N170 was quantified at lateralised electrodes P7, P8, TP9, and TP10. For the latter components (P1 and N170), which are characterized by a distinct peak, we extracted amplitude values within a time window of 20 ms around the component peak determined for each participant by averaging over all artefact-free trials within the respective ROI. P1 peaks were identified as most positive deflection in the time window of 90–150 ms after stimulus onset (mean peak latency = 135 ms); N170 peaks were defined as most negative deflection from 150 to 220 ms (mean peak latency = 191 ms). For all ERP components, artefact-free single-trial amplitudes were z-normalised across experimental conditions; on trials with artefacts, amplitude values were replaced with the respective mean (i.e., z-normalised amplitude of zero).

For the analyses of ERPs, component amplitudes of artefact-free trials were averaged per subject and experimental condition (Congruency (2) X Attention (2)) and analysed using a mixed-effects GLM, with the fixed factors Congruency and Attention and Subject as a random factor.

#### 2.5.4 Joint EEG-fMRI

Normalized (z-scored) single-trial ERP values for each ERP component and Congruency condition were convolved with the FSL canonical hemodynamic response function and included as parametric regressors in the fMRI analyses (8 additional regressors: C1, P1, N170 and P3 for congruent and incongruent trials). GLM analysis of fMRI data was identical to that described above, except for the addition of these parametric regressors. We then identified brain regions showing significant covariation of ERP amplitudes with BOLD response in the Attend Face condition, separately for each congruency condition. Since all ERP regressors were included in the same first level model, identified brain regions show trial-by-trial covariation of BOLD response *unique* to each ERP component, over and above mean condition-specific BOLD activation. However, since this analysis would not account for any variance that is *shared* between components, we also included additional contrasts for pairs of components (e.g., C1 + P1 congruent, etc.).

## 3 Results

### 3.1 Behaviour

RT analyses showed longer reaction times for trials where participants attended to faces compared to words, *F*(1,14.08) = 29.694, *p* < 0.001, *η_p_^2^* = 0.678. Furthermore, RTs were longer for incongruent than for congruent trials, *F*(1,14.37) = 103.49, *p* < 0.001, *η_p_^2^* = 0.878. A significant interaction between Attention and Congruency reflected a larger effect of congruency when participants attended to faces compared to words, *F*(1,14.63) = 51.31, *p* < 0.001, *η_p_^2^* = 0.778.

Accuracy rates were higher in the word condition than in the face condition, *F*(1,14.17) = 5.59, *p* < 0.05, *η_p_^2^* = 0.296, and for congruent compared to incongruent trials, *F*(1,14.30) = 37.01, *p* < 0.001, *η_p_^2^* = 0.721. A significant interaction between Attention and Congruency was based on a larger effect of Congruency in the face condition compared to the word condition, *F*(1,14.31) = 16.19, *p* < 0.001, *η_p_^2^* = 0.531. Together, RTs and accuracy indicate greater interference of conflicting information for the Attend Face condition than for Attend Word.

**Fig. 1:**
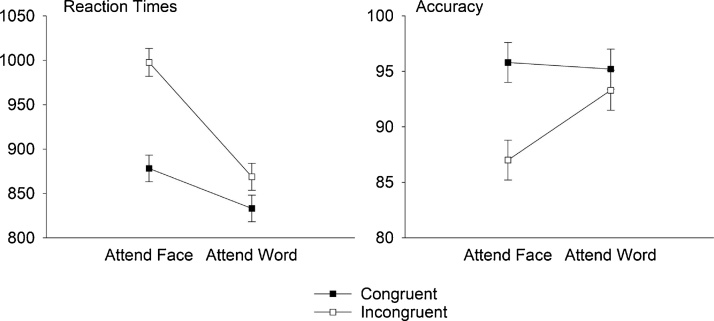
Means and 95% confidence intervals for RTs and accuracy rates.

Importantly for the attentional focus manipulation, there was a significant difference in reaction times between congruent trials in the Attend Face vs. Attend Word condition, *F*(1,14) = 7.16, *p* < 0.05, *η_p_^2^* = 0.34. Responses were slower for faces than for words in the congruent condition (i.e., when stimuli were identical), demonstrating the impact of the top-down attention manipulation. For RTs and accuracy rates, see and Fig. 1.

### 3.2 fMRI

**Fig. 2:**
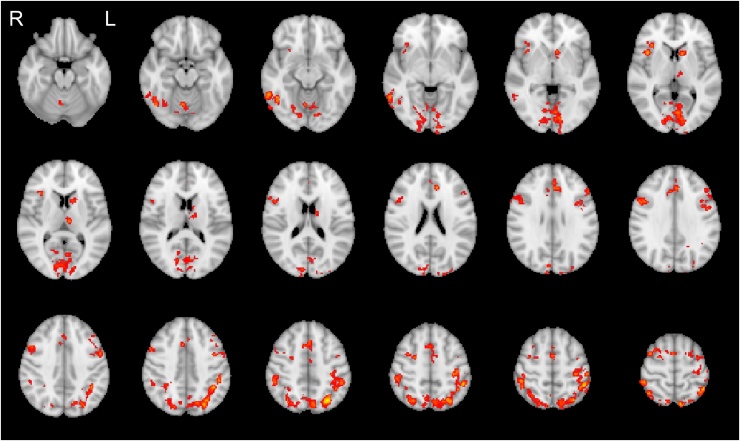
BOLD activations for incongruent − congruent trials. Significant activations were limited to the Attend Face condition. Activations are whole-brain corrected at FWE < 0.05.

When participants performed the categorisation task on the facial expressions (Attend Face), a number of brain regions showed significantly higher activation for incongruent than for congruent trials (see Fig. 2). These regions included large parts of the visual cortex, superior and middle frontal gyrus, and bilateral middle temporal cortex. For a complete list of significant activations see Table 1. In the Attend Word condition, there were no significant activation differences between congruent and incongruent trials.

**Table 1:** Summary of fMRI results from fMRI and joint EEG-fMRI analyses. Activations are whole-brain corrected at FWE < 0.05.

Condition	Brain regions	Local maxima (mm)			Cluster size (voxels)
		*X*	*y*	*z*	
fMRI Analyses					
Attend Face Congruent − Incongruent	L: middle temporal gyrus	−56	−56	6	255
	R: middle temporal gyrus, inferior temporal gyrus, superior temporal gyrus, supramarginal gyrus	48	−28	−6	311
	Paracingulate gyrus, anterior cingulate cortex, medial superior frontal gyrus	8	18	40	1430
	L: insula, frontal operculum, superior frontal gyrus, middle frontal gyrus, inferior frontal gyrus	−42	16	2	1994
	R: insula, frontal operculum, middle frontal gyrus, inferior frontal gyrus	40	14	2	2071
	Intracalcarine cortex, occipital pole, lingual gyrus	−12	−86	2	3262
	Bilaterally: lateral occipital cortex, superior parietal lobule, supramarginal gyrus, postcentral gyrus	−28	−66	50	3935

Joint EEG-fMRI Analyses (Attend Face Congruent)					
N170	L: lateral occipital cortex, angular gyrus	−50	−64	24	394
C1	Precuneous cortex	−2	−52	52	255
	L: orbitofrontal cortex, inferior frontal gyrus	−50	24	−8	311
	L: middle temporal gyrus	−68	−34	−4	378
	L: lateral occipital cortex, angular gyrus, middle temporal gyrus	−26	−68	30	830
	Posterior cingulate gyrus	4	−48	36	902

Control analyses: Shared variance (Attend Face Congruent)					
C1 + N170	L: lateral occipital cortex, angular gyrus	−42	−74	40	366
P1 + N170	L: lateral occipital cortex, angular gyrus	−44	−74	38	373
N170 + P3	R: middle frontal gyrus	30	20	58	261
	R: lateral occipital cortex, angular gyrus	40	−62	56	423
	L: lateral occipital cortex, angular gyrus	−40	−62	54	534
	Posterior cingulate cortex	0	−42	24	431

### 3.3 ERPs

**Fig. 3:**
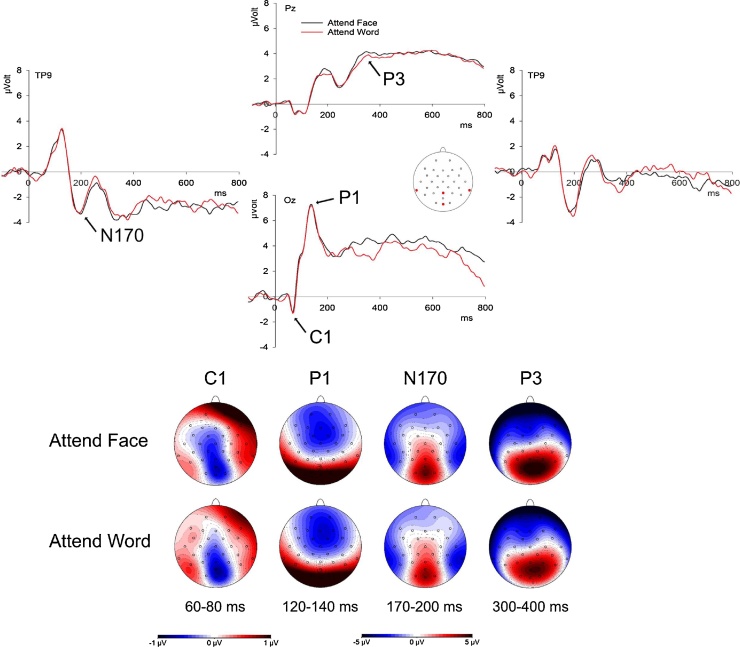
ERP waveforms and topographies. Mean ERP waveforms for Attend Face and Attend Word on four electrodes Pz, Oz, TP9 and TP10 (electrode positions are marked in red). In the lower half, scalp topographies for ERP components included in the joint EEG-fMRI analyses for the two Attention conditions at indicated time intervals. (For interpretation of the references to colour in this figure legend, the reader is referred to the web version of this article.)

Analyses revealed no significant effects of Attention or Congruency for the amplitudes of C1, P1, and N170. P3 amplitudes showed a main effect of Attention, *F*(1,14.31) = 17.748, *p* < 0.001, *η_p_^2^* = 0.554, based on larger P3 amplitudes when participants attended to faces compared to words (see Fig. 3).

### 3.4 Joint EEG-fMRI

The joint EEG-fMRI analyses including all ERP components (C1, P1, N170 and P3) as parametric regressors yielded significant results only for two ERP components, C1 and N170.

#### 3.4.1 N170

**Fig. 4:**
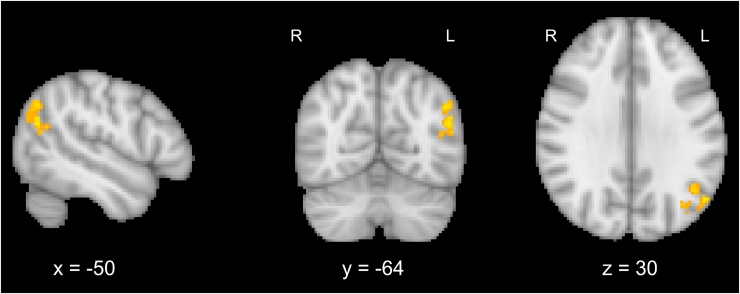
Trial-by-trial covariations with N170 amplitudes in the Attend Face condition in the left lateral parietal-occipital cluster (x = −50, y = −64, z = 30). Activations are whole-brain corrected at FWE < 0.05.

N170 amplitudes in the congruent attend face condition showed significant covariation with a region at the intersection of the lateral occipital cortex and angular gyrus (Fig. 4). No significant covariations were found for incongruent trials.

**Fig. 5:**
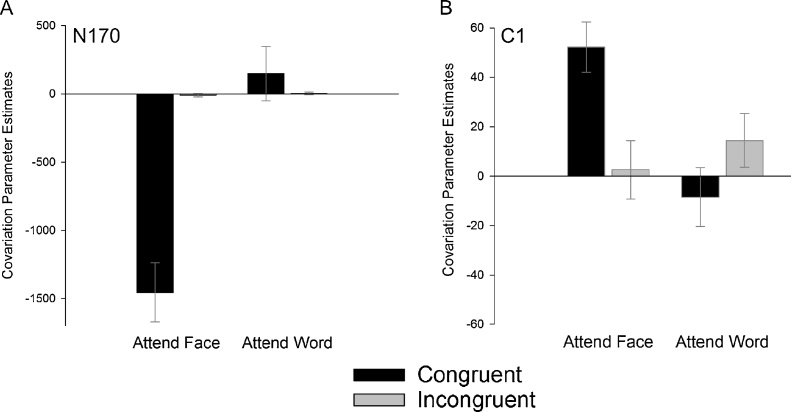
Covariation parameter estimates for Attention x Congruency conditions within significant clusters of covariation for the (A) N170 component and (B) C1 component. Error bars depict 95% confidence intervals.

In order to test whether this area showed selective coupling to faces in the congruent condition, we extracted covariation parameter estimates within this region for all four Attention x Congruency conditions and performed post-tests on covariation parameter estimates using a mixed effects GLM with Attention and Congruency as fixed factors and Subject as a random factor. Analyses showed main effects of Attention, *F*(1,14) = 18.42, *p* < 0.001, and of Congruency, *F*(1,14) = 6.14, *p* = 0.05, and an interaction between the two factors, *F*(1,14) = 19.22, *p* < 0.001, confirming that significant coupling was limited to congruent trials in the Attend Face condition (see Fig. 5A).

#### 3.4.2 C1

**Fig. 6:**
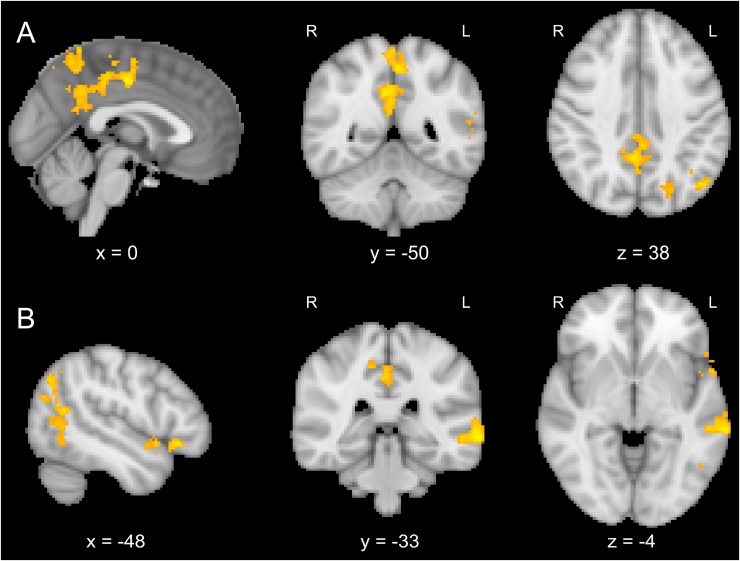
Brain regions showing covariation with amplitudes in the C1 time window (60–80 ms) for congruent trials in the Attend Face condition in A) precuneous cortex and posterior cingulate gyrus; B) left lateralized activations in a lateral occipital cluster (at the conjunction of lateral occipital cortex, middle temporal gyrus and angular gyrus), in the middle temporal gyrus and in the orbitofrontal cortex/inferior frontal gyrus. Activations are whole-brain corrected at FWE < 0.05.

When participants attended to the face in congruent trials, C1 amplitudes showed significant covariations with a number of brain regions, including the precuneous cortex and the posterior cingulate gyrus, as well as left-lateralized activations in the lateral parietal-occipital cluster described above, middle temporal cortex and temporal pole (see Fig. 6). Post-tests on covariation values revealed a main effect of Attention, *F*(1,14) = 5.28, *p* < 0.05, *η_p_^2^* = 0.274, and a significant interaction between Attention and Congruency, *F*(1,14) = 8.41, *p* < 0.05, *η_p_^2^* = 0.375, confirming that the coupling was limited to congruent trials in the Attend Face condition (Fig. 5B). In incongruent trials, analyses revealed no voxelwise covariations of C1 amplitudes in the Attend Face condition.

#### 3.4.3 Bold main effects in covariation clusters

In order to shed light on relations between ERP-BOLD covariation and BOLD activation itself, we extracted BOLD parameter estimates within brain regions showing significant covariations with ERP amplitudes, separately for Congruency and Attention conditions. We performed a mixed effects GLM with the fixed factors Attention, Congruency and cluster, and Subject as random factor. For the N170 cluster, there were no significant differences in BOLD activation between experimental conditions. For brain regions showing significant coupling with C1 amplitudes in the Attend Face condition, analyses revealed significantly more BOLD activation for incongruent compared to congruent trials, *F*(1,14) = 6.26, *p* < 0.05, *η_p_^2^* = 0.309.

#### 3.4.4 Shared variance in EEG-fMRI analyses

**Fig. 7:**
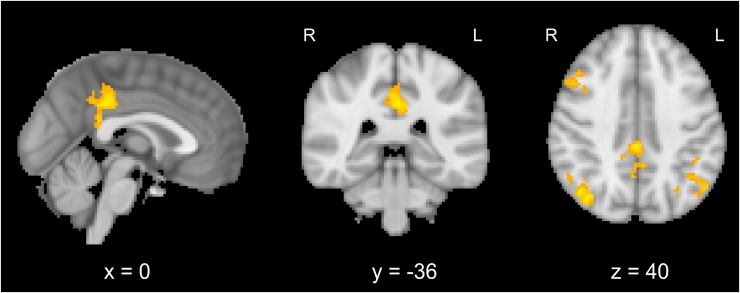
Covariations with amplitudes of N170 and P3 (shared variance) in the Attend Face condition in the lateral parietal-occipital cluster (bilaterally), posterior cingulate gyrus and middle frontal gyrus. Activations are whole-brain corrected at FWE < 0.05.

The EEG-fMRI analyses included additional contrasts for pairs of components in order to account for possible shared variance between components. Results showed additional covariations in the lateral parietal-occipital cluster (see Table 1) between C1 + N170 and P1 + N170 components in the congruent condition, corresponding to the brain area reported above. N170 and P3 showed shared variance in a number of brain regions, including significant bilateral covariations in the lateral parietal-occipital cluster. Further regions were located in the posterior cingulate cortex and the middle frontal gyrus (see Fig. 7). Notably, all significant shared covariations were again limited to the congruent condition.

## 4 Discussion

Using simultaneous EEG-fMRI during a face-word emotional Stroop task, the present study investigated the influence of visual cortex activity on higher-order face processing stages, and its top-down modulation by task-related attention. We predicted that, in the Attend Face condition, early ERP components would covary from trial to trial with early and later stage face processing regions, and that this covariation would be reduced or absent during the Attend Word condition. We also made a nondirectional prediction of an effect of word-face congruency on ERP-fMRI covariation. We found that trial-by-trial ERP amplitudes within 80 ms of stimulus onset showed significant covariations with a widespread network of brain regions. Importantly, these covariations were limited to congruent trials in the Attend Face condition, showing their dependence on sustained attentional focus and modulation by trial-by-trial conflict processing.

When participants performed emotion categorisations on faces while ignoring superimposed words (Attend Face condition), the ERP amplitudes of the C1 window extending from 60 to 80 ms after stimulus onset showed significant covariations with a widespread network of brain regions, including a lateral parietal-occipital cluster and left-lateralized activations of the orbitofrontal cortex, middle temporal gyrus and inferior frontal gyrus. The lateral parietal-occipital cluster was located at the intersection of the superior lateral occipital cortex and angular gyrus, extending into posterior middle temporal gyrus. This region has previously been associated with a number of functions including face processing (Leveroni et al., 2000, Nielson et al., 2010), semantic processing, social cognition and memory retrieval, and was suggested as an integration zone within the default mode network (Andrews-Hanna, Reidler, Sepulcre, Poulin, & Buckner, 2010; Greicius, Krasnow, Reiss, & Menon, 2003; Seghier, 2012). The more anterior brain regions are associated with emotion processing (Adolphs, 2002, Rolls and Grabenhorst, 2008, Sabatinelli et al., 2011) and semantic processing (Noonan, Jefferies, Visser, & Lambon Ralph, 2013). Both the lateral parietal-occipital cluster and anterior regions may comprise part of an extended network that adds emotion and meaning to perceived faces (Gobbini and Haxby, 2007, Leveroni et al., 2000). C1 window covariations also appeared in the precuneus and the posterior cingulate gyrus, brain regions involved in attention orientation and focus on the self, including autobiographical processing (Cavanna & Trimble, 2006; Leech & Sharp,2014). The covariations of these brain regions with trial-wise C1 amplitudes reveal that variations in very early activity in the visual cortex have a measureable influence on higher-order cognitive functions that add meaning to visually presented faces.

Importantly, however, and despite identical visual input, covariations in this brain network only occurred when participants attended to face stimuli, and not when words were the targeted stimuli. This result indicates a strong influence of task-related top-down attentional focus on covariation between early visual processing as measured with ERPs and the extended face processing network. Top-down attentional demands can modulate early-stage visual processing, including the primary visual cortex, influenced by factors such as attention, reward, and mood (Bayer et al., 2016, Proverbio et al., 2010, Rossi and Pourtois, 2012, Vanlessen et al., 2013). Yet our results reveal the effect of attentional focus on the *coupling* of early ERP amplitudes with a more broadly distributed network of brain regions, but not with primary visual cortical regions. This result is consistent with top-down attention acting as a “gate”, permitting or preventing early visual cortical processing (which did not itself show modulation in this task) to flexibly influence higher order processing depending on task demands. Previous ERP research has shown spatial attentional gating of ERPs to emotional faces at frontal cortical sites between 100 and 120 ms post stimulus-onset (Holmes, Vuilleumier, & Eimer, 2003). Our results indicate that top-down attention might gate even earlier stages of visual processing.

In addition, we found that C1 ERP-fMRI covariations were limited to congruent trials within the Attend Face condition, showing their sensitivity to trial-wise conflict processing and the impact of conflicting information. Our data revealed a stronger Stroop effect, i.e. the difference between congruent and incongruent trials in RTs and accuracy, within the Attend Face condition, showing greater interference of (unattended) words on face processing than vice versa, in line with assumptions that reading occurs automatically whenever a word is presented (Posner and Snyder, 1975, Stroop, 1935). Similarly, fMRI results showed that significant differences in BOLD activation between congruent and incongruent trials were limited to the Attend Face condition. Consistent with previous literature, incongruent trials in the Attend Face condition elicited higher activation in brain regions associated with top-down control (Botvinick, Cohen, & Carter, 2004; Dosenbach et al., 2007). One tentative explanation for the lack of ERP-fMRI covariation for incongruent Attend Face trials is that conflict-related up-regulation of activation (see also Banich et al., 2001) in these areas in case of incongruent trials might override the impact of sensory information.

Amplitudes of the N170 component showed specific covariation with activation in the lateral parietal-occipital cluster during congruent trials in the Attend Face condition, a brain region associated with semantic or emotional information about faces (Jehna et al., 2011, Leveroni et al., 2000). The N170 has previously been linked to structural encoding of faces and face identity processing (Eimer, 2000, Eimer et al., 2011), but in this case did not show covariations with brain regions associated with these processes such as the fusiform gyrus. One tentative suggestion is that our task did not focus on face identification, but on variable aspects of faces (cf. Haxby et al., 2000). Beyond that, it is notable that covariations revealed in our analyses generally seemed to occur in brain regions associated with higher-order processing rather than lower-level sensory areas.

Results of our control analyses on conjoint component pairs were largely in line with results from our main analyses, showing significant covariations in the left lateral parietal-occipital cluster. Furthermore, conjoint N170 + P3 covariations were located bilaterally in the lateral parietal-occipital cluster and in the posterior cingulate cortex. Finally, the latter component pair showed significant covariations with the middle frontal gyrus, which is suggested to play a role in reorienting attention between the dorsal and the ventral attention network (Corbetta, Patel, & Shulman, 2008; Japee, Holiday, Satyshur, Mukai, & Ungerleider, 2015). As in previous analyses on unique covariations, significant results were limited to the congruent condition.

We employed EEG-informed fMRI analyses by making use of trial-wise, condition-specific ERP amplitudes for multiple ERP components related to face processing, starting from processing in the (extra)striate visual cortex up to higher-order stimulus evaluation, with multiple ERP components included in the same GLM. The major advantage of this approach is its power to reveal *unique*, i.e. condition-specific, and, more importantly, component-specific covariations, while also allowing for the full use of the high temporal resolution of the EEG signal. At the same time, additional control analyses on conjoint component pairs are employed to reveal potential shared variance between components. Interestingly, and in line with previous literature (Sadeh, Podlipsky, Zhdanov, & Yovel, 2010), condition-specific covariations occurred irrespective of ERP effects. Although C1 and N170 amplitudes did not show significant differences between experimental conditions, they still showed significantly different covariations. This highlights the complementary value of combined ERP-fMRI analysis, which uses trial-by-trial variation as a potentially informative measure of neural processing.

The most striking finding of this study was that trial-by-trial variation of visual processing of faces as early as 60–80 ms after stimulus onset showed unique covariation with a widespread network of brain regions, indicating the impact of early stage visual processing on higher-order functions, including semantic and emotional processing. Crucially, this covariation was modulated by the sustained attentional focus (on faces versus words) and depended on trial-by-trial conflict processing, suggesting a flexible and reciprocal link between top-down and bottom-up processing.

## Funding

This work was supported by BBSRC (grant No. BB/H011021/1 to TJ), the Berlin School of Mind and Brain, Humboldt-Universität zu Berlin (MB), and EPSRC (EP/J500501/1 to MTR).
